# Noise from the periphery in autism

**DOI:** 10.3389/fnint.2013.00034

**Published:** 2013-07-24

**Authors:** Maria Brincker, Elizabeth B. Torres

**Affiliations:** ^1^Philosophy Department, University of MassachusettsBoston, MA, USA; ^2^Movement Disorders, Neurology Department, Indiana University School of MedicineIndianapolis, IN, USA; ^3^Psychology Department, Rutgers Center for Cognitive Science, Center for Computational Biomedicine Imaging and Modeling (Computer Science), Movement Disorders, Neurology, Rutgers University School of Medicine, Rutgers UniversityNew Brunswick, NJ, USA

## The disembodied-static brain approach to ASD

No two individuals with the autism diagnosis are ever the same—yet many practitioners and parents can recognize signs of ASD very rapidly with the naked eye. What, then, is this phenotype of autism that shows itself across such distinct clinical presentations and heterogeneous developments? The “signs” seem notoriously slippery and resistant to the behavioral threshold categories that make up current assessment tools. Part of the problem is that cognitive and behavioral “abilities” typically are theorized as high-level disembodied and modular functions—that are assessed discretely (impaired, normal, enhanced) to define a spectral syndrome. Even as biology reminds us that organic developing bodies are not made up of independent switches, we remain often seduced by the simplicity of mechanistic and cognitive models. Developmental disorders such as autism have accordingly been theorized as due to different modular dysfunctions—typically of cortical origin, i.e., failures of “theory of mind” (Baron-Cohen et al., 1985), of the “mirror neuron system” (Ramachandran and Oberman, 2006), of “weak central coherence” (Happe and Frith, 2006) or of the balance of “empathizing” and “systemizing” (Baron-Cohen, 2009), just to list a few.

The broad array of autonomic (Ming et al., 2005; Cheshire, 2012) and sensorimotor (Damasio and Maurer, 1978; Maurer and Damasio, 1982; Donnellan and Leary, 1995; Leary and Hill, 1996; Donnellan and Leary, 2012; Donnellan et al., 2012) differences experienced and reported by people with autism have by such theories typically been sidelined as “co-morbidities,” possibly sharing genetic causes, but rendered as incidental and decisively behaviorally irrelevant symptoms—surely disconnected from cognition. But what if the development of cortically based mental processes and autonomous control relies on the complexities and proper function of the peripheral nervous systems? Through such an “embodied” lens the heterogeneous symptoms of autism invites new interpretations. We propose here that many behavioral-level findings can be re-defined as downstream effects of how developing nervous systems attempt to cope and adapt to the challenges of having various noisy, unpredictable, and unreliable peripheral inputs.

Self-advocates have long tried to describe their unique phenomenological experiences—and many talk about not being able to trust, feel, or control their bodies as they would intentionally prefer. Many tell us that parts of their bodies seem to disintegrate experientially, that sensory stimulations are either too intensely invading or go unnoticed, entirely collapsing into each other as echoes (Savarese, 2007; Robledo et al., 2012; Amos, 2013). Such experiences of living and coping with ASD, along with the widespread reports of sensorimotor and autonomic differences have led us to explore the hypothesis that individuals with autism are coping with unreliable peripheral signals from atypically self-organized subsystems. On the basis of recent sensorimotor findings (Torres et al., 2013) discussed below we speculate that various kinds of peripheral noise result in unpredictability of the person's movements and their re-afferent kinesthetic proprioception. These in turn impede central coordination and autonomous control, and force the developing system to find alternative avenues of prediction and anticipatory control.

## Sensing through movement—not all variability is created equal

What do we mean by noise? Noise might be defined as any kind of sensed phenomenon or change that cannot be interpreted as a signal (Kosko, 2006). Thus, the idea of noise instantly craves a discussion of how we interpret or make sense of the stochastic world that impinges on all our afferent nerves at any point in time; aka the riddle of sense perception that has haunted natural philosophers since antiquity. How can we, with a body in constant motion, get to a coherent and stable perception of anything? The scientific and philosophical world is starting to wake up to the idea that this riddle must be solved through understanding the dynamics of predictive anticipation not only of own body position and motion in time, but also the contents of what is perceived (Friston, 2012). But how do we do this if our movements are always inherently variable—even when trying to reproduce the same movement? (Bernstein, 1967).

What have often been overlooked are the processes and relevance of continuously accumulating evidence from the fluctuations in our motions. By gaining a *probabilistic expectation about the variability itself*, the system can acquire predictable and reliable “motor priors.” Rather than merely adding “noise” (Faisal et al., 2008), sensory-motor variability can serve as actively sampled and sharpened informative “signals” and as an aid in adaptively reshaping old priors.

## Corrupted motor priors in ASD

Using a new statistical platform for behavioral analyses (SPBA) (Torres and Jose, 2012) a new study has begun the experimental estimation of the idiosyncratic patterns of movement variability unique to each person. By itself a single fluctuation in our motions (micro-movement) is not informative. However, when looked at as stochastic processes, the continuous flow of our natural behaviors reveal the family of probability distributions best describing the degrees of predictability and reliability in the behavioral variability of each person. Applying the SPBA, one can see that the statistical properties of our behavior undergo maturation. Typically developing (TD) children begin to gain reliable and predictable re-afferent feedback of limb micro-movements around 4 years of age. By college age, young graduates manifest even more predictable and reliable patterns with a broader bandwidth of values (Torres et al., 2013). However, none of the 34 subjects with ASD (ages 4–25)—independent of verbal proficiency and gender—showed predictive micro-movements. Rather their fluctuations were random and “memoryless.” The speed-dependent variability from prior trials was not more predictive of future trials than was the variability from a current trial. In this sense the movement variability from experiencing the “here and now” seemed to be the only useful kinesthetic information to them. Moreover, the bandwidth of speed values was very narrow in ASD, despite their ability to reach the goals of the task.

People with matured “motor priors” can learn to sense the statistics of the impinging world “through” their own movement fluctuations. When predictable and reliable, these serve as malleable anchors to adaptively help separate internal from external influences and enable the system discriminate intended from spontaneous variations (Torres, 2013). Given the acquired ability to integrate the sensed local motor expectations and other sensed influences, the overall background enables unexpected sensed re-afferent variability to fluidly morph from *noise* to perceptual *signal* rather flexibly, as any new situation might require.

People with autism have goal-directness, but their re-afferent feedback (kinesthetically sensed though their movements fluctuations) fails to establish reliable probabilistic expectations of their own movement variations. Lack of motor priors impedes acquisition of baselines to build an embodied perceptual foundation. Without such a frame of reference to assess new contextual variations as signals, every variation and contextual influence intensifies the noise already inherent in the movement. Accordingly it could be interesting to empirically explore a possible connection between absence of baseline motor expectation and difficulties with, for example, cross-modal integration (Iarocci and McDonald, 2006). Mapping their own physical movement variations onto those of others in the social scene must be difficult for the person with autism—a tractable hypothesis using the new SPBA (Johnson et al., 2012). Under the kinesthetic re-afference hypothesis we can begin to understand social withdrawal or timidity as a coping response to the intense uncertainty and loss of control that social situations must produce in the person with autism.

We should stress that the absence of reliable “motor priors” in ASD does not give us the causes of autism. However, it helps begin to define the challenges in new inclusive ways, where the affected person is part of the solution. By precisely and objectively quantifying movement sensing in autism, we can begin to develop an operational definition that refines our understanding and offers tractable routes of behavioral intervention, even when the causes are unknown. This definition will not merely enumerate what is different or deficient in the autistic system relative to what is known in the typical system. It will, instead, harness whatever compensatory-adaptive solution the autistic system has already developed and work with that to help steer their performance toward social-communicative goals.

These findings can be seen as complementing the intense world syndrome theory (Markram et al., 2007; Markram and Markram, 2010). Movement variability could also help define the phenotype in animal essays by bridging experimental manipulations at the molecular level with precise measurements of behavioral outcomes showing their intense manifestations.

The model that we propose conceives sensory-motor exchange as a dynamic-stochastic process, whereby the noise-to-signal ratios evolve in the system as behavior unfolds over time with non-stationary statistics (Torres, 2013; Torres et al., 2013). We can track the shifts in stochastic signatures with precise statistical indexes of reliability and predictability in real-time. We can also measure the bandwidth of values that each person has access to through the re-afferent kinesthetic information. Thus, we are in a position to tackle the heterogeneity of ASD. Progress can be evaluated to determine the rates of change of their stochastic trajectories and to track the changes in the signs of the derivative of this process to *experimentally* construct optimal vs. sub-optimal scenarios in real situations. Performance can then be steered by closing the stochastic sensory-motor feedback loops to selectively co-adapt the autistic system with the type of sensory guidance that recruits, modulates, and enhances central autonomy over the body. This would then allow us to tap into many of the solutions that the autistic system has already self-discovered. Their system can show us the optimal path of least resistance [in a very precise physical sense (Lanczos, 1966; Feynman et al., 2006)]: the path that accelerates learning. In this regard our model is by definition inclusive of the individual with ASD.

## Separable anatomy and time scales of proprioceptive development

The empirical evidence discussed above involves the rather slow maturation of the limbs and hands sensory-motor variability. However, phylogenetic evolution and specialization of the facial and hand muscles differ in time and order of appearance, as suggested by the cytoarchitectonics of the cerebral cortex (Allman, 1999; Mountcastle, 2005). It is thus our proposition that the atypical trajectories in maturation of motor priors accompanied by a consistent embodied differentiation between central and peripheral influences may manifest and be detectable much earlier in the stochastic signatures of facial micro-expressions. The latter are supported by important cranial nerves that innervate orofacial muscles critical for survival in neonates (Porges, 2003). Functions include suckling, swallowing, developing movement patterns to assist mastication later on, and generally coordinating sound production and reception to communicate distress or pleasure to the progenitor. Emotional content and autonomic regulations delivered by the active configuration and spontaneous relaxation of these muscles critically depend on the feedback-loops involving these nerves (Bazhenova et al., 2007; Field and Diego, 2008). To understand the full range of autistic symptoms we must, as proposed by Porges, look at the dynamics between phylogentically distinct but interacting systems (Porges et al., 1994; Bazhenova and Porges, 1997; Porges, 2003). Understanding and objectively quantifying movement fluctuations as a form of re-afferent kinesthetic input in neurotypical infants may lead us to earlier detection of critical aberrancies potentially leading to neurodevelopmental differences with complex downstream regulatory consequences.

In conclusion, we propose to shift the almost exclusive focus on cortical issues in autism to the issues in the peripheral nervous systems and their dynamic contribution to the heterogeneity of the disorder. In so doing, we will be able to non-invasively quantify these differences in real-time during therapeutic interventions, drug treatments, and natural behaviors in general. The adaptive progress in each person can be tracked, and this can help sort out genetic or traumatic causes and assist in the development of personalized therapies. Such therapies will be driven by objective real-time quantification of noise as the autistic system shows us how it transforms it and steers it into predictable and reliable signals for anticipatory autonomous control.

It is time that we seek to better understand how the distributed intelligence of our bodies and social environments scaffolds our cortical control functions for self-autonomy. The measurable re-afferent micro-movements can help us track the dynamics of embodied minds and thereby also *move* autism research, diagnoses and treatments toward a new frontier—one that includes and truly connects us with the most important piece of this puzzle: the individual with autism.

## References

[B1] Allman J. M. (1999). Evolving Brains. New York, NY: Scientifc American Library; Distributed by W.H. Freeman and Co.

[B2] Amos P. (2013). Rhythm and timing in autism: learning to dance. Front. Integr. Neurosci. 7:27 10.3389/fnint.2013.00027 23626527 PMC3630367

[B3] Baron-Cohen S. (2009). The empathizing–systemizing (E-S) theory. Ann. N.Y. Acad. Sci. 1156, 68–80 10.1111/j.1749-6632.2009.04467.x 19338503

[B4] Baron-Cohen S. Leslie A. M. Frith U. (1985). Does the autistic child have a “theory of mind”? Cognition 21, 37–46 10.1016/0010-0277(85)90022-8 2934210

[B5] Bazhenova O. V. Porges S. W. (1997). Vagal reactivity and affective adjustment in infants. Convergent response systems. Ann. N.Y. Acad. Sci. 807, 469–471 10.1111/j.1749-6632.1997.tb51940.x 9071371

[B6] Bazhenova O. V. Stroganova T. A. Doussard-Roosevelt J. A. Posikera I. A. Porges S. W. (2007). Physiological responses of 5-month-old infants to smiling and blank faces. Int. J. Psychophysiol. 63, 64–76 10.1016/j.ijpsycho.2006.08.008 17056142 PMC1790728

[B7] Bernstein N. (1967). The Co-Ordination and Regulation of Movements. Oxford: Oxford Press

[B8] Cheshire W. P. (2012). Highlights in clinical autonomic neuroscience: new insights into autonomic dysfunction in autism. Auton. Neurosci. 171, 4–7 10.1016/j.autneu.2012.08.003 23040840

[B9] Damasio A. R. Maurer R. G. (1978). A neurological model for childhood autism. Arch. Neurol. 35, 777–786 10.1001/archneur.1978.00500360001001 718482

[B10] Donnellan A. M. Hill D. A. Leary M. R. (2012). Rethinking autism: implications of sensory and movement differences for understanding and support. Front. Integr. Neurosci. 6:124 10.3389/fnint.2012.00124 23372546 PMC3556589

[B11] Donnellan A. M. Leary M. R. (1995). Movement Differences and Diversity in Autism/Mental Retardation: Appreciating and Accommodating People with Communication and Behavior Challenges. Madison, WI: DRI Press

[B12] Donnellan A. M. Leary M. R. (2012). Autism: Sensory-Movement Differences and Diversity. Cambridge, WI: Cambridge Book Review Press

[B13] Faisal A. A. Selen L. P. Wolpert D. M. (2008). Noise in the nervous system. Nat. Rev. Neurosci. 9, 292–303 10.1038/nrn2258 18319728 PMC2631351

[B14] Feynman R. P. Leighton R. B. Sands M. L. (2006). The Feynman Lectures on Physics. San Francisco, CA: Pearson/Addison-Wesley

[B15] Field T. Diego M. (2008). Vagal activity, early growth and emotional development. Infant Behav. Dev. 31, 361–373 10.1016/j.infbeh.2007.12.008 18295898 PMC2556849

[B16] Friston K. (2012). Prediction, perception and agency. Int. J. Psychophysiol. 83, 248–252 10.1016/j.ijpsycho.2011.11.014 22178504 PMC3314993

[B17] Happe F. Frith U. (2006). The weak coherence account: detail-focused cognitive style in autism spectrum disorders. J. Autism Dev. Disord. 36, 5–25 10.1007/s10803-005-0039-0 16450045

[B18] Iarocci G. McDonald J. (2006). Sensory integration and the perceptual experience of persons with autism. J. Autism Dev. Disord. 36, 77–90 10.1007/s10803-005-0044-3 16395537

[B19] Johnson G. Yanovich P. Difeo G. Yang L. Santos E. Ross N. (2012). What do we see in each other: how movement drives social interaction, in IGERT-NSF Video and Poster Competition: Award Winning, (Washington, DC).

[B20] Kosko B. (2006). Noise. New York, NY: Viking

[B21] Lanczos C. (1966). The Variational Principles of Mechanics. Toronto, ON: University of Toronto Press

[B22] Leary M. R. Hill D. A. (1996). Moving on: autism and movement disturbance. Ment. Retard. 34, 39–53 8822025

[B23] Markram H. Rinaldi T. Markram K. (2007). The intense world syndrome–an alternative hypothesis for autism. Front. Neurosci. 1, 77–96 10.3389/neuro.01.1.1.006.2007 18982120 PMC2518049

[B24] Markram K. Markram H. (2010). The intense world theory – a unifying theory of the neurobiology of autism. Front. Hum. Neurosci. 4:224 10.3389/fnhum.2010.00224 21191475 PMC3010743

[B25] Maurer R. G. Damasio A. R. (1982). Childhood autism from the point of view of behavioral neurology. J. Autism Dev. Disord. 12, 195–205 7174608 10.1007/BF01531309

[B26] Ming X. Julu P. O. Brimacombe M. Connor S. Daniels M. L. (2005). Reduced cardiac parasympathetic activity in children with autism. Brain Dev. 27, 509–516 10.1016/j.braindev.2005.01.003 16198209

[B27] Mountcastle V. B. (2005). The Sensory Hand: Neural Mechanisms of Somatic Sensation. Cambridge, MA: Harvard University Press

[B28] Porges S. W. (2003). The Polyvagal Theory: phylogenetic contributions to social behavior. Physiol. Behav. 79, 503–513 10.1016/S0031-9384(03)00156-2 12954445

[B29] Porges S. W. Doussard-Roosevelt J. A. Maiti A. K. (1994). Vagal tone and the physiological regulation of emotion. Monogr. Soc. Res. Child Dev. 59, 167–186 7984159

[B30] Ramachandran V. S. Oberman L. M. (2006). Broken mirrors: a theory of autism. Sci. Am. 295, 62–69 17076085 10.1038/scientificamerican1106-62

[B31] Robledo J. Donnellan A. M. Strandt-Conroy K. (2012). An exploration of sensory and movement differences from the perspective of individuals with autism. Front. Integr. Neurosci. 6:107 10.3389/fnint.2012.00107 23162446 PMC3499780

[B32] Savarese R. J. (2007). Reasonable People: A Memoir of Autism and Adoption: on the Meaning of Family and the Politics of Neurological Difference. New York, NY: Other Press

[B33] Torres E. B. (2013). Signatures of movement variability anticipate hand speed according to levels of intent. Behav. Brain Funct. 9, 10 10.1186/1744-9081-9-10 23497360 PMC3635904

[B34] Torres E. B. Brincker M. Isenhower R. W. Yanovich P. Stigler K. A. Nurnberger J. I. (2013). Autism: the micro-movement perspective. Front. Integr. Neurosci. 7:32 10.3389/fnint.2013.00032 PMC3721360 23898241

[B35] Torres E. B. Jose J. V. (2012). Novel Diagnostic Tool to Quantify Signatures of Movement in Subjects with Neurobiological Disorders, Autism and Autism Spectrum Disorders. US patent application. New Brunswick, NJ: Office of Technology Commercialization, Rutgers, The State University of New Jersey

